# Immediate Transfusion

**Published:** 1872-11

**Authors:** 


IMMEDIATE TRANSFUSION.—Dr. J. H. Aveling, physician to the Chelsea Hospital for Women, reports a case to the *London Lancet*, of a woman who would certainly have died from post-partum hœmorrhage, due to inversion of the uterus, but for a timely resort to the operation of transfusion. The patient had no perceptible pulse in the radial and temporal arteries; was completely exhausted, and was insensible, and the iris was unaffected by the action of light; the extremities were cold, and the lips blanched. A vein at the bend of the patient's arm was exposed and opened, and the afferent tube inserted, which was imperfectly performed at first, though afterwards properly effected; the arm of a coachman was then ligatured, as in venesection, a vein opened, and the afferent tube inserted. The man was seated in a chair by the bed of the patient, and the rubber portion of the apparatus, filled with water, being now attached to the tubes. Transfusion then began, and was continued until more than sixty drachms of blood were injected. The patient recovered.

